# Effects of an 8-Week Cooperative Learning Intervention on Physical Education Students’ Task and Self-Approach Goals, and Emotional Intelligence

**DOI:** 10.3390/ijerph18010061

**Published:** 2020-12-23

**Authors:** Sergio Rivera-Pérez, Javier Fernandez-Rio, Damián Iglesias Gallego

**Affiliations:** 1 Physical Education & Exercise Lab, Teacher Training College, University of Extremadura, 10003 Cáceres, Spain; sergioriveraperez@gmail.com (S.R.-P.); diglesia@unex.es (D.I.G.); 2 Department of Education Sciences, Faculty of Teacher Training and Education, University of Oviedo, 33003 Oviedo, Spain

**Keywords:** cooperative learning, achievement goals, emotional intelligence, physical education

## Abstract

Previous research highlighted the effectiveness of cooperative learning in the four learning domains: physical, cognitive, social and affective. However, recent reviews have called for more empirical research on social and emotional learning based on contemporary theories, frameworks and assessment tools. Little is known about the links between cooperative learning and two strong contemporary frameworks: the achievement goal theory and the four-branch model of emotional intelligence. The goal of this study was to assess the connections between cooperative learning, task and self-approach goals, and emotional intelligence in physical education classes. Forty primary education students (21 girls, 19 boys), 10–12 years (*M_age_* = 10.87; *SD* = 0.85), enrolled in two different classes in only one school, participated. None of them had experienced cooperative learning as a pedagogical model before. The study followed a one group, pre-test-post-test, pre-experimental design. Both classes experienced the same cooperative learning intervention programme conducted in physical education, which included two consecutive learning units for a total of 16 sessions (2 per week/50 min each). The same physical education teacher, an expert in cooperative learning, conducted all sessions. Results showed that the cooperative learning framework helped increase students’ self-approach goals and their emotional control and regulation, and empathy. In conclusion, the present study reinforced the use of cooperative learning in physical education, because it can guide students to more adaptive motivational patterns and to develop their emotional intelligence. Furthermore, it contributes to the students’ social and emotional learning building quality relationships, learning to manage stressors, and evolve individually and in groups.

## 1. Introduction

Teaching/instruction in physical education has evolved rapidly over the last 50 years from the spectrum of teaching styles [1] to teaching models [2], curricular models [3], instruction models [4], and the current pedagogical models [5,6]. These are student-centred, but also closely consider context, content, teaching and learning [7,8,9]. Cooperative learning is considered a pedagogical model [10]. 

In cooperative learning contexts, students work in small, heterogeneous groups with, from and for other students, sharing resources and efforts to maximize their own and their groupmates’ learning, with students and teachers considered co-learners [9,11,12,13]. Several authors agree that cooperative learning includes the following five basic elements [14,15,16]: (a) interpersonal skills: students must listen to each other, share ideas, give and receive feedback, take turns, encourage each other; (b) group processing: students reflect and discuss during and after the different tasks to assess their functioning; (c) positive interdependence: students learn that they can only have success if the other group mates are successful too; (d) individual accountability: students must contribute to the group’s goal, since their individual contribution is necessary for the group’s success; and (e) promotive interaction: students work in direct contact with each other during the different tasks. In a recent review of cooperative learning implementation in physical education, Bores-García et al. [17] found that it can contribute to students’ motor [18,19], social [20,21,22], physical [23,24], cognitive [25,26] and affective learning [27]. Regarding social and emotional learning, in a recent scoping critical review, Dyson et al. [28] made a call for more empirical research on “the attainment of SEL [Social and Emotional Learning] students outcomes in PE [Physical Education] using a contemporary theory, framework and recognized assessments” (p.14) to develop a common understanding. In the present study, two important theoretical frameworks have been used to uncover the links between cooperative learning and two social and emotional learning outcomes: emotional intelligence and achievement goals.

Emotional intelligence is considered a socio-affective construct that refers to the individuals’ capacity to understand and manage emotions and, consequently, use them in appropriate situations and contexts [29]. Within this construct, three frameworks have been identified: (1) The ability model or four-branch model, which is based on the identification, understanding, regulation, expression and usage of one’s and others’ emotions [30]; (2) The model of emotional competencies, which believes that there is a number of competences that facilitate the acknowledgment and management of emotions [31,32]; and (3) The model of emotional and social intelligence, which believes that emotional learning is a group of emotional, personal and social knowledge and skills that affect individuals’ ability to face the stressful situations that surrounds them [33].

The present study followed the four-branch model, because it is the most widely used and there are internationally validated assessment instruments [34]. Within this framework, elements like self-regulation, self-knowledge, empathy and social/interpersonal skills have been connected to satisfactory group work [35]. Unfortunately, group work does not come naturally (individuals do not know how to work within a group effectively), and regulation within groups can shift from one group member adopting a leading role to a more co-regulatory context where all group members share leadership [36]. Previous research in educational contexts found positive connections between emotional intelligence and the development of positive social skills [37], higher academic achievement and better psychosocial adjustment [38].

Due to its socio-affective character, emotional intelligence fits in a construct that is receiving increasing attention: social and emotional learning. It has been defined as the process that helps individuals learn and manage a number of social and emotional skills necessary to succeed in contexts where intrapersonal relationships occur [39]. It also means the use of cognitive regulation and emotional processing [40]. In a previously mentioned review, Dyson et al. [28] indicated that cooperative learning can promote individuals’ social and emotional learning in physical education contexts, because this pedagogical model promotes the development of social/interpersonal and emotional skills. Social learning has been more frequently researched in cooperative learning, particularly students-teachers’ connections and motivation [22,41], while emotional learning remains under researched. In a previous study on emotional learning variables, Luca and Tarricone [35] found that when group members have adequate emotional intelligence, two key elements in cooperative learning, positive interdependence and promotive interaction, increase. More recently, in a cross-sectional study, a positive association was found between cooperative learning and emotional intelligence in all school stages during physical education classes [42]. This finding suggested a positive connection between both frameworks. Unfortunately, research is still limited, especially in physical education contexts, and more studies are needed to fill gaps in the scientific literature: can cooperative learning make an impact in students’ emotional learning? Can it promote students’ empathy or emotional regulation? Essential elements like group processing or individual accountability point in the positive direction, but research should answer these questions.

One of the most relevant theories used to evaluate students’ motivational process in the context of physical education is the achievement goal theory [43]. It is rooted in the social-cognitive theory of motivation and it deals with the type of goals that individuals adopt when performing an activity. Initially, only two goals were identified [44]: (1) Performance or trying to show competence relative to others, and (2) Mastery or trying to show competence relative to a task. This framework evolved into a trichotomous model [45] when performance goals were divided into approach goals, focusing on being positive to have success, and avoidance goals, focusing on avoiding failure. Over the years, mastery goals were also divided and the 2 × 2 goal model was developed [46]. It included four goals: (1) Mastery-approach or being positive focusing on the task, (2) Mastery-avoidance or avoiding performing the task poorly, (3) Performance-approach or focusing on outperforming others, and (4) Performance-avoidance or avoiding performing worse than others. Finally, Elliot et al. [47] introduced the 3 × 2achievement goal framework and three distinctive standards: task-based, self-based (both originated from the original mastery goals) and others-based (from the performance goals). Task-based goals adopt an absolute reference: the task; self-based goals set an intrapersonal reference: oneself; finally, other-based use an interpersonal or normative reference: other individuals or a norm [48]. Based on this framework, the 3 × 2 achievement goals model [47] includes six goals that rise from the three standards used to define competence (task, self, other) and the two previously mentioned valences (approach and avoidance): (1) Task-approach goals focus on achieving competence on the task (i.e., performing a task correctly); (2) Task-avoidance goals focus on avoiding incompetence on the task (i.e., “performing better than before”); (3) Self-approach goals focus on achieving competence based on oneself (i.e., “performing better than before”); (4) Self-avoidance goals focus on avoiding incompetence based on oneself (i.e., “avoiding performing worse than before”); (5) Other-approach goals focus on achieving competence based on others (i.e., “performing better than others”); and (6) Other-avoidance goals focus on avoiding incompetence based on others (i.e., “avoiding performing worse than others”).

González-Cutre et al. [49] found that teachers can create a mastery-oriented class climate with positive effects on students’ mastery achievement goals. In a recent review, Liu et al. [50] described that: “goals aimed at mastering knowledge and developing self-competence lead to favourable consequences. If goals orient people to compete against others or avoid normative failure, consequences can be detrimental” (p. 294). Previous research found a connection between task and self-approach goals and students’ intrinsic motivation [47,51]. In this same trend, García-González et al. [52] showed that task-oriented climates promote the most self-determined types of motivation (i.e., intrinsic motivation). As previously mentioned, in cooperative learning contexts, students work in small, heterogeneous groups to maximize their own and their groupmates’ learning, not to outperform others [9,11,13]. Therefore, they could be considered mastery-oriented contexts and they have been found to be able to promote students’ intrinsic motivation [20,53,54]. To our knowledge, the connections between students’ achievement goals and cooperative learning have not been studied.

Almost a couple of decades ago, Elliot [55] warned that mastery-approach goals can produce adaptive outcomes and achievement goals, while mastery-avoidance goals were linked to less adaptive outcomes. Later, Elliot et al. [47] widened the scope to inform that approach goals produced more adaptive responses than avoidance goals. Recent research conducted in physical education contexts found that task-approach goals and self-approach goals produced adaptive responses in students’ self-determined motivation and life satisfaction [56]. However, globally, the highest adaptive pattern was observed in the task-approach goals, which should be promoted in physical education [57]. Given the recommendation to focus on task-approach goals for positive learning outcomes, the current study used task and self-approach goals in a physical education setting. Further, as cooperative learning includes working with others to master a task, individually and with the group, cooperative learning as a pedagogical model was followed. As previously mentioned, cooperative learning is about working with others to master a task, both individually and within a group. Therefore, students’ task-approach and self-approach goals should be promoted when using this pedagogical model. Little is known about the behavioural consequences of cooperative learning contexts on students’ achievement goals and emotional learning. Therefore, the aim of this study was to assess the effects of an eight-week cooperative learning intervention on physical education students’ task and self-approach goals, and emotional intelligence. Based on theoretical predictions [10,47], as well as previous research [56,57], we build two hypotheses. The first one was that students’ task and self-approach goals will increase after experiencing cooperative learning. The second hypothesis was that students’ emotional recognition, emotional control and regulation, and emotional empathy will increase after experiencing cooperative learning. 

## 2. Methods

### 2.1. Participants and Setting 

A one group, pre-test-post-test pre-experimental design and convenience sampling were used to recruit participants [58]. A total of 40 primary education students (21 girls, 19 boys), age range 10–12 years (*M_ag_*_e_ = 10.87; *SD* = 0.85), enrolled in two different classes (20 in year five, 20 in year six) in one Primary School located in western Spain agreed to participate. None of them had experienced cooperative learning as a pedagogical model before. The school was placed in an urban context and children belonged to families with a medium-low socioeconomic status. The intervention program was conducted by one physical education teacher, an expert in cooperative learning. Zeni [59] argues that conditions of classic experimental research, such as random selection and control groups, are irrelevant and problematic for insider teacher research. As such, there were no control groups in the present study.

### 2.2. Intervention Programme

According to Hastie and Casey [60], fidelity of model implementation should be addressed through: (a) a rich description of the curricular elements of the unit, (b) a detailed validation of model implementation and (c) a detailed description of the program context (p. 243). 

In regards to the detailed description of the program context, both classes experienced the same intervention programme based on cooperative learning. It lasted 8 weeks for a total of 16 physical education sessions (2 per week/50 min each). The same physical education teacher conducted all sessions on both classes. Some members of the research team were experts in cooperative learning, which included more than 15 years of theory, practice and research on this pedagogical model. They were in direct contact with the participating teacher to design the intervention programme. They closely supervised all tasks and lessons to make sure that the learning units adhered to the model’s framework [14]. Moreover, the research team met regularly on-line with the participating teacher to support, discuss and provide feedback, shaping a participatory action research project [61].

Regarding the curricular elements of the unit, each session was designed to include the five key elements of cooperative learning [16]: interpersonal skills, group processing, positive interdependence, promotive interaction and individual accountability (Table 1). The first learning unit, “Let’s get to know our bodies”, included eight sessions focused on body awareness, body management, movement concepts, respiration-relaxation, balance and coordination. The second learning unit, “cooperative motor skills”, included another eight sessions focused on locomotor skills, throwing and catching.

In each session, several cooperative learning techniques or structures were used: (1) learning teams [62], based on learning together [16]: students worked in groups of four and each one played a role (teacher performer, observer, equipment manager), changing roles every few attempts to learn a new skill (i.e., forward roll). (2) Co-op play [63]: students had to cooperate to achieve a group goal to work on coordination (i.e., all group members had to cross the gym stepping only inside rings). (3) Pairs-check-perform [62], based on pairs check [63]: students worked in pairs to improve a locomotor skill (i.e., jump over different obstacles across a space), one acted as a performer and the other as a teacher giving instructions (they shifted roles after a few tries). (4) Think-share-perform [62], based on think-pair-share [63]: students faced challenges to work on throwing and catching skills (i.e., throw and catch while moving under/over obstacles) which forced them to share ideas, negotiate, think and try the different solutions to solve the challenge. (5) Collective score [64]: together, the class tried to solve a challenge to obtain a “class score” (i.e., pass the ball striking it by hands to one another during one minute); the class had several tries to share ideas and beat the score. (6) Learning groups [65], based on learning teams [16]: students worked in small groups of four to improve their locomotor skills (i.e., crawling, leaping, skipping over/under an obstacle course) playing only two roles: three were performers and one the coach that provided feedback. 

Finally, regarding the validation of model implementation, one member of the research team, an expert in cooperative learning, observed one out of two sessions to provide feedback and supervise the correct implementation. In addition, an internationally validated questionnaire on students’ perceptions of a cooperative learning context was included in the study [66]. It provided information on the (un)successful implementation of the model to create a true cooperative learning context. 

### 2.3. Procedure

First, the Bioethics and Biosafety Committee of the University of Extremadura approved the study. Second, the participating school was contacted, the project was fully explained to the administrators and permission was obtained. Only then, it was introduced to the students and their parents, and these were asked to sign a written consent. All procedures respected the ethical principles of research on human beings [67] and data obtained was treated following recommendations of the American Psychological Association, which included confidentiality and anonymity. Data were collected prior and right after the intervention programme was conducted. Participating students were asked to answer honestly, because the answers were kept confidential and they would not influence their physical education class grades. Both data collection sessions were conducted in the students’ regular classroom to provide a safe atmosphere.

### 2.4. Data Collection 

#### 2.4.1. Cooperative Learning Questionnaire

Ref [66]. It is an internationally validated instrument that assesses the essential components of cooperative learning in educational contexts. In the present study, it was headed by the stem: “In my Physical Education classes…”. It includes 20 items grouped in five factors: interpersonal skills (i.e., “We listen to each other’s ideas, opinions and points of view”); group processing (i.e., “We talk to each other to make sure that everyone in the group knows what is being done”); positive interdependence (i.e., “We cannot finish the tasks without the groupmates’ contributions”); promotive interaction (i.e., “Interaction among groupmates is necessary to complete the tasks”); and individual accountability (i.e., “It is important for every group member to try to participate, even if he/she does not like the task”). This instrument also provides a global cooperation factor, obtained from the mean score of the five subscales. Participants responded in a 5-point Likert scale from one: “Totally disagree” to five: “Totally agree”. Previous research identified acceptable reliability and validity in this instrument [66]. For the present study, adequate Cronbach’s alphas were obtained for all factors: interpersonal skills (0.72), group processing (0.76), positive interdependence (0.73), promotive interaction (0.78), individual accountability (0.82) and global cooperation factor (0.90).

#### 2.4.2. 3 × 2 Achievement Goals Questionnaire in Physical Education 

Ref [57]. The Spanish validated version for physical education contexts of the original questionnaire developed by Elliot et al. [47] was used. Because of the goals of the study, only two subscales were used: task-approach (i.e., “perform correctly many tasks”) and self-approach (i.e., “perform the tasks better than I have done them before”). The questionnaire begins with the stem: “In my Physical Education classes, my goal is to...”. Participants responded in a 5-point Likert scale from one: “Totally disagree” to five: “Totally agree”. Previous research identified acceptable reliability and validity in this instrument [57]. In the present study, Cronbach’s alpha values were: 0.88 for task-approach goals and 0.87 for self-approach goals.

#### 2.4.3. Emotional Intelligence Questionnaire in Physical Education 

Ref [34]. It includes 22 items grouped in three factors: emotional recognition (i.e., “I know when I am getting angry during exercises and/or games”), emotional control and regulation (i.e., “I feel totally relaxed”), and emotional empathy (i.e., “I manage effectively my classmates’ anger”). The scale begins with the stem: “In my Physical Education classes...”. Participants responded in a 5-point Likert scale from one: “Totally disagree” to five: “Totally agree”. In the original study, Cronbach’s alphas were between 0.88 (emotional control and regulation, and emotional empathy) and 0.90 (emotional recognition). Previous research identified acceptable reliability and validity in this instrument [34]. In the present study, Cronbach’s alpha values were: 0.86 (emotional recognition), 0.88 (emotional control and regulation) and 0.91 (emotional empathy).

### 2.5. Data Analyses

The sample size and the normality tests recommended the use of non-parametric tests (Shapiro-Wilk test). Therefore, U Mann-Whitney tests were conducted to assess gender and/or grade differences in pre and post-intervention scores. The Wilcoxon-Rank test was used to assess pre-post changes.

## 3. Results

First, no significant differences (*p* > 0.05) were found at pre-tests in any of the observed variables regarding gender. Second, no significant differences (*p* > 0.05) were found post-intervention in any of the observed variables regarding gender. 

Based on the previously mentioned results, all subjects were grouped in a single study group. The Wilcoxon-Rank test (Table 2) uncovered a significant increase in self-approach goals, emotional control and regulation, and emotional empathy at post-test. In all cases, the effect size (Cohen’s *d*) was moderate. Marginally significant increases (tendency) (*p* = 0.060) were observed for task-approach goals. Finally, significant increases were observed in all the elements of cooperative learning and in the global cooperation factor with a large effect size.

## 4. Discussion

The aim of this study was to assess the effects of an eight-week cooperative learning intervention on physical education students’ task and self-approach goals, and emotional intelligence. Results showed that the cooperative learning framework helped increase students’ self-approach goals and their emotional control and regulation, and empathy.

All the basic elements of cooperative learning significantly increased from pre to post-tests, which indicates that the framework was successfully implemented. Recent reviews warned against a lack of rigor in some of the implementations conducted [17], which failed to include all the cooperative learning basic elements or a validation of the model implementation, and the shortness and fragmentation of the experiences conducted [10]. The present study included the five basic elements of cooperative learning and the intervention program could be considered long (eight weeks/16 lessons). Moreover, a recent study showed that when cooperative learning is not highly structured, it does not yield the same positive outcomes [68]. This is very important, because research needs to “demonstrate” that the results obtained are a direct consequence of the framework used. In the present study, all basic cooperative learning elements increased from pre to post-tests, showing that the model was adequately used and the outcomes could be considered derived from it.

Regarding the first hypothesis that students’ task and self-approach goals will increase after experiencing cooperative learning, results only partially confirmed it. Students’ self-approach goals significantly increased, while their task-approach goals only showed a positive tendency (not a statistically significant change). This statistically significant increase connects directly with cooperative learning’s theoretical framework, and it reinforces the idea that when it is correctly implemented, students’ individual accountability increases and, consequently, their self-approach goals. It suggests that the intervention helped students focus on their part of the group’s task, on achieving competence based on oneself [“performing better than before”; [47], in this case to contribute to the groups’ success. This could be considered a very positive and adaptive element for students’ learning and development, promoted by the cooperative framework. To our knowledge, this is the first study to assess the possible connections of cooperative learning and students’ achievement goals, and it shows that cooperative learning can increase individuals’ self-approach goals, which, in turn, benefit the group. There is a need for more research to support or reject this idea and deepen in this connection. Previous research has hypothesized that the direct connection between cooperative learning and achievement goals would be through task-approach goals [57], which means focusing on achieving competence on the task [“performing a task correctly”; [47]. However, in highly structured [68], properly co-regulated cooperative working structures [36], each individual is responsible for one part of the group’s task, who must focus on “performing better than before” (self-approach). The significant increase found in this achievement goals, added to the significant increase in the students’ individual accountability support this view. 

Results partially support the second hypothesis, such that emotional control and regulation, as well as emotional empathy, increased after experiencing cooperative learning; emotional recognition did not change. These findings suggest that cooperative learning can promote students’ emotional intelligence and it is in line with recent cross-sectional research that found this same positive association [42]. In the present study, both basic elements and students’ emotional control and regulation, and emotional empathy significantly increased at post-test, which reinforces the connection between the two frameworks (cooperative learning and emotional intelligence). When students work in direct contact with each other during the different tasks (promotive interaction) and learn that they can only have success if the other group mates are successful too (positive interaction) they understand or feel what another person is experiencing (empathy) and are better able to control their emotional state (emotional control and regulation). A few years earlier, Luca and Tarricone [35] discussed that basic cooperative learning elements such as individual accountability and group processing could promote individuals’ emotional regulation and empathy. Results from the present study partially confirms this idea, since the emotional regulation increased after the students experienced cooperative learning. Previous research has also indicated that emotional control and regulation increases through social relations and group work [10,68], two basic elements of cooperative learning that significantly increased at post-tests. Recently, Dyson et al. [28] found that the learning outcomes of this pedagogical model align and complement students’ social and emotional learning. Results from the present study confirms this idea, legitimizing popular views on the connections between both frameworks. 

The present study holds some strengths. It is the first empirical study on the connections between cooperative learning and the 3 × 2 achievement goal model. It is also the first intervention study to assess the impact of this pedagogical model on students’ emotional intelligence. Furthermore, fidelity to model implementation has been fully assessed through an internationally validated tool and significant increases have been verified. Therefore, the findings from the present study could be considered a substantial contribution to the scientific and pedagogical knowledge on the behavioural consequences linked to the use of high-structured, properly co-regulated cooperative learning. However, the present study also holds some limitations, being the first one with the absence of a control group (controversial causality). The second one is the limited number of participants and the fact that they all belonged to the same school. Therefore, results should be confirmed in future studies conducted in larger groups of students, enrolled in different schools and using control groups to compare.

## 5. Conclusions

Results from the present study indicated that cooperative learning, when implemented in physical education, can help increase students’ self-approach goals. This could be considered a noteworthy contribution to the scientific and pedagogical literature on cooperative learning model, since it can produce a shift in the students towards more adaptive motivational patterns. A second significant contribution was that the present study strengthened the links between cooperative learning and students’ emotional intelligence. This pedagogical model seemed to help them understand or feel what another person is experiencing and control their emotional state when working in groups. Results from the present study reinforced the use of cooperative learning in physical education, because it can contribute to the students’ social and emotional learning building quality relationships, learning to manage stressors, and evolving as individuals and as a group [69,70].

## Figures and Tables

**Table 1:** Key cooperative learning elements in each unit.

Key Elements	Unit 1	Unit 2
Interpersonal skills	Students encouraged each other	Students deliberately shared resources
Group processing	Students reflected individually and together	Students shared ideas to solve the tasks
Positive interdependence	The tasks ended when all the classmates have completed it	Students switched from one activity to another when all members were ready
Promotive interaction	Students interacted during the tasks to finish them	Students performed the tasks in direct contact with each other
Individual accountability	Each student performed his/her role during the tasks	Each student performed his/her part of the task

**Table 2:** Means, standard deviations and Wilcoxon rank test.

Scales	Variables	Pre-Test	Post-Test	Z	*p*-Value	Effect Size
**CLQ**	Interpersonal skills	3.47 ± 0.96	4.05 ** ± 0.84	−3.237	0.001	0.52
	Group processing	3.47 ± 0.96	4.08 ** ± 0.77	−3.794	0.000	0.61
	Positive interdependence	3.86 ± 0.67	4.22 ** ± 0.68	−3.234	0.001	0.52
	Promotive interaction	3.87 ± 0.66	4.17 ** ± 0.68	−2.822	0.005	0.45
	Individual accountability	4.02 ± 0.73	4.28 * ± 0.68	−2.236	0.025	0.36
	Global cooperation factor	3.74 ± 0.59	4.16 ** ± 0.61	−4.441	0.000	0.71
**3 × 2 AGQ-PE**	Task-approach goals	4.20 ± 0.83	4. 39 ± 0.66	−1.882	0.060	0.30
	Self-approach goals	4.08 ± 0.80	4.34 * ± 0.77	−1.973	0.048	0.32
**EIQPE**	Emotional recognition	4.12 ± 0.62	4.20 ± 0.64	−1.071	0.284	0.17
	Emotional control and regulation	3.65 ± 0.67	3.97 * ± 0.60	−2.346	0.019	0.38
	Emotional empathy	3.57 ± 0.63	3.98 ** ± 0.66	−3.728	0.000	0.60

Note: * *p* < 0.05; ** *p* < 0.01.

## Data Availability

Data is contained within the article.
